# The distribution of axial length, anterior chamber depth, lens thickness, and vitreous chamber depth in an adult population of Shahroud, Iran

**DOI:** 10.1186/1471-2415-12-50

**Published:** 2012-09-18

**Authors:** Hassan Hashemi, Mehdi Khabazkhoob, Mohammad Miraftab, Mohammad Hassan Emamian, Mohammad Shariati, Tahereh Abdolahinia, Akbar Fotouhi

**Affiliations:** 1 Noor Ophthalmology Research Center, Noor Eye Hospital, Tehran, Iran; 2 Farabi Eye Hospital, Tehran University of Medical Sciences, Tehran, Iran; 3 Shahroud University of Medical Sciences, Shahroud, Iran; 4 Department of Community Medicine, School of Medicine, Tehran University of Medical Sciences, Tehran, Iran; 5 Department of Optometry, Tehran University of Medical Sciences, Tehran, Iran; 6 Department of Epidemiology and Biostatistics, School of Public Health, Tehran University of Medical Sciences, Tehran, Iran

**Keywords:** Axial length- Anterior chamber depth lens thickness- Vitreous chamber depth, Normal range

## Abstract

**Background:**

Ocular biometric parameters can be influenced by race, ethnicity, and genetics; their differences across different populations can probably explain differences in refractive errors in these populations. The aim of this study is to determine the normal range of axial length, anterior chamber depth, lens thickness, and vitreous chamber depth in the population of Shahroud in the north of Iran.

**Methods:**

In the first phase of Shahroud Eye Cohort Study, the 40–64 year old population were sampled cross-sectionally; 6311 were invited and 5190 (82.2%) participated in the study. Biometric examinations were done using the LENSTAR**/**BioGraph (WaveLight AG, Erlangen, Germany) after vision tests and before cycloplegic refraction tests. Any type of eye surgery, extensive pterygium, and lack of cooperation were used as exclusion criteria, and analyses were done with data from 4869 eyes.

**Results:**

We found a mean axial length of 23.14 mm (95% confidence interval [CI], 23.11-23.17), mean anterior chamber depth of 2.62 mm (95% CI, 2.60-2.63), mean lens thickness of 4.28 mm (95% CI, 4.27-4.29), and the mean vitreous chamber depth was 15.72 mm (95% CI, 15.70-15.75).

Kolmogorov-Smirnov tests showed that the distribution of axial length, anterior chamber depth, lens thickness, and vitreous chamber depth significantly differed from normal; axial length and vitreous chamber depth demonstrated a leptokurtic distribution as well.

Axial length, anterior chamber depth, and vitreous chamber depth significantly decreased with age, and lens thickness significantly increased with age (p < 0.001). All indices were significantly higher in men.

**Conclusions:**

The distributions of axial length, vitreous chamber depth, and lens thickness are reported for the first time in an Iranian adult population. Compared to other studies, axial length was in the mid range, nonetheless, studying axial length components showed that the Iranian population had smaller anterior chamber depth and lens thickness. Age and gender were significantly associated with all indices assessed in this study.

## Background

Global advances in ophthalmology have created a greater need for ocular parameters in different clinical and diagnostic fields. One important ophthalmic parameter is the axial length (AL) which is commonly needed for intraocular lens power calculation before cataract and refractive surgery [1] and helps ophthalmologists in the diagnosis of several eye conditions such as staphyloma, [2] and risk of retinal detachment [3].

In addition to clinical applications, determining ocular biometry, especially the AL and its components in epidemiologic studies, provides ophthalmologists with important and valuable information. Reports concerning the distribution of ocular biometrics in population based studies have been published from some Asian countries such as Mongolia, Taiwan, Myanmar, Singapore and China [4-8].

Several studies have demonstrated the correlation between ocular biometrics, especially AL, with refractive errors [6,9]. Since these parameters can be influenced by race, ethnicity, and genetics, their differences across different populations can probably explain differences in refractive errors, and it would be useful to determine the distribution of biometric indices in each area.

There are few studies on the distribution of biometrics, especially axial length, in normal populations in the Middle East region and Iran [9,10]. As a population-based study, the report from Saudi Arabia [10] has the limitation of a small sample size. The study on Jordanian adults [9] only targeted the 17 to 40 year old population, and the study by Yekta et al. [11] in Iran showed ocular biometrics in carpet weavers where a considerable proportion were myopic and their results cannot be generalized to the normal population. Here we report the distribution of AL and its components including the anterior chamber depth (ACD), lens thickness (LT), and vitreous chamber depth (VCD) in a general 40–64 year old Iranian population.

## Methods

Data of the present study was derived from the first phase of the Shahroud eye cohort study, which was conducted cross-sectionally in 2009. In brief, samples of the study were selected from the 40–64 year old population of Shahroud using random cluster sampling where 300 clusters from 9 strata (health care centers) of Shahroud city were randomly selected. From each cluster, 20 people were invited to have complete eye examinations.

At the time of the 2006 census, the population of Shahroud was 133835. Of these, 28779 were in the 40–64 year old age group; 14720 (51.1%) of which were men. In this group, 29.0% were 40–44 years old, 26.8% were between 45 and 49 years of age, 20.3% were 50–55, 13.8% were 55–59, and 10.1% were between 60 and 64 years old. In this study, 6311 people were selected from this population.

All consenting participants were first interviewed to record their demographics, socio-economic status, as well as their medical and ophthalmic history. For ophthalmologic examinations, people were examined with the slit lamp biomicroscope, and if no contraindication existed for cyclopentolate eye drops, they had cycloplegic refraction tests.

### Biometry

All participants had ocular biometry tests with the LENSTAR**/**BioGraph (WaveLight AG, Erlangen, Germany) after vision testing and before ophthalmologic examination and cycloplegic refraction. The Biograph generates different ocular biometry indices and here the AL, ACD, LT, and corneal thickness readings were used. Acquisitions were done by a skilled operator who was trained before the study. The validity and repeatability of LENSTAR/BioGraph measurements have been confirmed before [12,13], and thus, one acquisition was made per eye.

### Definitions and statistical analysis

Since VCD is not measured directly with the Biograph, ACD, LT, and corneal thickness (mm) values were deducted from AL to calculate VCD. Each ocular biometry index is described as mean and 95% confidence intervals (CI) by age and gender, and the normal range calculated as the mean ± 2 standard deviations. the 25^th^, 50^th^, 75^th^, 95^th^ and 99^th^ percentiles were determined to demonstrate the distribution of these variables in more detail. The relationship of AL and its components with age, gender, height, weight, and education was studied with univariate linear regression, as well as with multivariate regression after adjusting variables. The t-test was used to compare the mean age of the participants and non-participants, and the chi-square test was used to examine differences in gender distribution between the selected sample and the respondents. To assess distribution differences from normal, distributions were plotted on histograms after using the Kolmogorov-Smirnov test. Pearson correlation coefficients were determined to examine the correlation between two eyes in terms of AL and its components.

In this report, only data from phakic eyes were included for analysis, and those who had any history of eye surgery were excluded. The correlation between left and right eyes was high in case of the indices of AL (r = 0.880), ACD (r = 0.958), LT (r = 0.880), and VCD (r = 0.931), and thus, only results from right eyes and presented here.

### Ethical considerations

Before examinations, and after providing a detailed description of the study and its methodology, all participants signed written informed consents. The study was reviewed and approved by the Ethics Committee of Shahroud University of Medical Sciences.

## Results

The invitees of Shahroud Eye Cohort Study were 6311 people and 5190 responded (82.2%). The mean age of the participants and non-participants was 50.9 years and 50.6 years, respectively, and their difference was not statistically significant (p = 0.160).

Of the participants, we excluded 151 people due to history of ocular surgery or history of ocular trauma (115 people cataract surgery, 7 people glaucoma surgery, 8 people retinal surgery, and 21 people due to a history of ocular trauma). Data of 170 people was not considered due to lack of cooperation, presence of extensive pterygium, or receiving an error message from the device. Eventually, analysis was done on 4869 eyes. Of this sample, 2825 (58%) were women; 97.6% were of Persian ethnicity, 2.0% were Turk, and 0.4% were non-Persian and non-Turk although none of the ethnic groups are a different race and all considered Middle Eastern.

In the studied sample, mean AL was 23.14 mm (95% CI, 23.11 - 23.17), mean ACD was 2.62 mm (95% CI, 2.60 - 2.63), mean LT was 4.28 mm (95% CI, 4.27 - 4.29), and the mean VCD was 15.72 mm (95% CI, 15.70 - 15.75).

**Figure 1:**
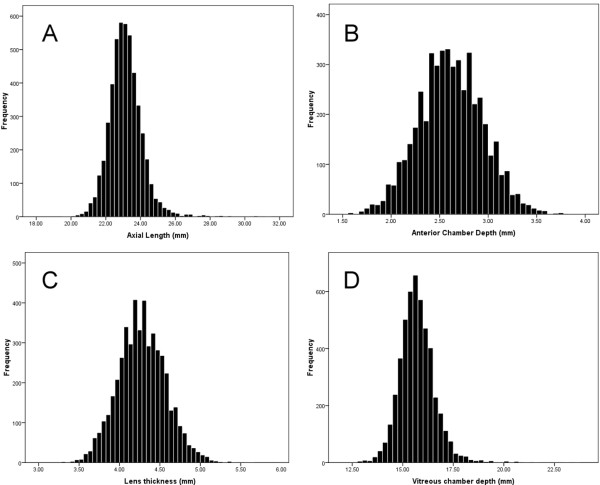
Distribution of axial length (A), anterior chamber depth (B), lens thickness C), and vitreous chamber depth (D).

Results in terms of mean and 95% CI of mean of the AL, ACD, LT, and VCD in the studied population by age and gender, the mean ± 2 SD of these variables by age and gender, and the 1^th^, 5^th^, 25^th^, 50^th^, 95^th^ and 99^th^ percentiles of these variables are summarized in Tables 1, 2, and 3, respectively. Figure 1 shows the histogram of the distribution of AL, ACD, LT, and VCD. Kolmogorov-Smirnov tests indicated a significant difference from normal distribution (p < 0.001). Table 3 summarizes the skewness and kurtosis of the variables. According to indices of normal distribution, axial length and vitreous chamber depth had leptokurtic distribution.

**Table 1:** Distribution of axial length (AL), anterior chamber depth (ACD), lens thickness (LT) and vitreous chamber depth (VCD) as mean and 95% confidence intervals of mean (CI) by age and gender

		**AL (mm)**	**ACD (mm)**	**LT (mm)**	**VCD (mm)**
**Age**	**n**	**Mean (95%CI)**	**Mean (95%CI)**	**Mean (95%CI)**	**Mean (95%CI)**
**40-44**	917	23.24 (23.17-23.30)	2.74 (2.72-2.76)	4.11 (4.09-4.13)	15.86 (15.80-15.93)
**45-49**	1332	23.16 (23.11-23.21)	2.66 (2.65-2.68)	4.22 (4.20-4.23)	15.75 (15.70-15.80)
**50-54**	1218	23.16 (23.10-23.21)	2.60 (2.58-2.62)	4.31 (4.30-4.33)	15.72 (15.67-15.78)
**55-59**	880	23.07 (23.00-23.13)	2.52 (2.50-2.54)	4.39 (4.37-4.41)	15.63 (15.57-15.70)
**60-64**	522	23.04 (22.96-23.12)	2.48 (2.45-2.51)	4.47 (4.44-4.50)	15.56 (15.49-15.64)
**Gender**					
**Male**	2044	23.41 (23.37-23.46)	2.66 (2.64-2.67)	4.30 (4.29-4.32)	15.93 (15.89-15.97)
**Female**	2825	22.95 (22.91-22.98)	2.58 (2.57-2.60)	4.26 (4.25-4.27)	15.58 (15.54-15.61)
**Total**	4869	23.14 (23.11-23.17)	2.62 (2.60-2.63)	4.28 (4.27-4.29)	15.72 (15.70-15.75)
**Valid data**		4833	4849	4840	4823

**Table 2:** The range (mean ± 2 standard deviations) of axial length (AL), anterior chamber depth (ACD), lens thickness (LT) and vitreous chamber depth (VCD) by age and gender

**Age**	**AL (mm)**	**ACD (mm)**	**LT (mm)**	**VCD (mm)**
**40-44**	21.29-25.19	2.12-3.35	3.59-4.63	14.02-17.71
**45-49**	21.25-25.07	2.02-3.31	3.69-4.75	13.96-17.54
**50-54**	21.22-25.10	1.97-3.23	3.76-4.86	13.90-17.55
**55-59**	21.05-25.08	1.86-3.18	3.83-4.95	13.75-17.52
**60-64**	21.17-24.91	1.81-3.15	3.87-5.07	13.76-17.36
**Male**	21.06-24.84	1.93-3.24	3.68-4.85	13.77-17.38
**Female**	21.53-25.30	1.99-3.33	3.71-4.90	14.14-17.72
**Total**	21.20-25.09	1.95-3.28	3.69-4.87	13.89-17.56

**Table 3:** The percentiles, Skewness, Kurtosis and interquartile range (IQR) of axial length, anterior chamber depth, vitreous chamber depth and lens thickness in this study

	**Percentile**							**Normal distribution indexes**		
	**1%**	**5%**	**25%**	**50%**	**75%**	**95%**	**99%**	**Skewness**	**Kurtosis**	**IQR**
**Axial length**	21.06	21.72	22.55	23.08	23.65	24.64	26.11	1.18	5.93	1.10
**Anterior chamber depth**	1.85	2.08	2.39	2.61	2.84	3.17	3.41	0.10	0.02	0.45
**Lens thickness**	3.63	3.80	4.08	4.27	4.47	4.77	5.01	0.17	0.21	0.39
**Vitreous chamber depth**	13.78	14.42	15.17	15.66	16.20	17.16	18.52	1.24	6.32	1.03

The relationship of studied variables with age, gender, education, height, and weight was studied in univariate and multivariate regression models and results are summarized in Tables 4 and 5. According to the multivariate model, AL decreased with age, and directly correlated with the male gender, years of education, height, and weight. ACD also decreased by 0.013 mm per year of aging, and directly correlated with the male gender, years of education, height, and weight. This is while LT increased by 0.018 mm per year of aging; it was, on average, 0.026 mm larger in men compared to women, and the correlation with education and weight was reverse. VCD showed a statistically significant decrease of 0.011 mm per year while it significantly increased with more education, height, and weight.

**Table 4:** The association of axial length and its components with age and gender according to univariate linear regression

		**Coefficient (95%CI of coefficient)**	**P-value**
**Axial length (mm)**	Age (years)	−0.010 (−0.015 to −0.006)	<0.001
	Sex (male/female)	0.466 (0.41 to 0.522)	<0.001
	Education (Each year in school)	0.026 (0.021 to 0.031)	<0.001
	Height (cm)	0.033 (0.03 to 0.036)	<0.001
	Weight (Kg)	0.013 (0.01 to 0.015)	<0.001
**Anterior Chamber depth (mm)**	Age (years)	−0.014 (−0.015 to −0.012)	<0.001
	Sex (male/female)	0.076 (0.057 to 0.095)	<0.001
	Education (Each year in school)	0.008 (0.006 to 0.01)	<0.001
	Height (cm)	0.007 (0.006 -0.008)	<0.001
	Weight (Kg)	0.004 (0.003 to 0.005)	<0.001
**Lens thickness (mm)**	Age (years)	0.018 (0.017 to 0.02)	<0.001
	Sex (male/female)	0.040 (0.024 to 0.057)	<0.001
	Education (Each year in school)	−0.005 (−0.007 to −0.003)	<0.001
	Height (cm)	0.00 (−0.001 to 0.001)	0.678
	Weight (Kg)	−0.002 (−0.002 to −0.001)	<0.001
**Vitreous chamber depth (mm)**	Age (years)	−0.015 (−0.019 to −0.01)	<0.001
	Sex (male/female)	0.351 (0.299 to 0.403)	<0.001
	Education (Each year in school)	0.022 (0.017 to 0.027)	<0.001
	Height (cm)	0.027 (0.024 to 0.03)	<0.001
	Weight (Kg)	0.01 (0.008 to 0.012)	<0.001

CI = confidence interval.

**Table 5:** The association between axial length and its parameters with age and gender in multivariate linear regressions

		**Coefficient (95%CI of coefficient)**	**P-value**
**Axial length (mm)**	Age (years)	−0.007 (−0.012 to −0.003)	<0.001
	Sex (male/female)	0.111 (0.035 to 0.186)	0.004
	Education (Each year in school)	0.011 (0.005 to 0.016)	<0.001
	Height (cm)	0.025 (0.021 to 0.029)	<0.001
	Weight (Kg)	0.005 (0.003 to 0.007)	<0.001
**Anterior Chamber depth (mm)**	Age (years)	−0.013 (−0.014 to −0.012)	<0.001
	Sex (male/female)	0.038 (0.012 to 0.064)	0.004
	Education (Each year in school)	0.004 (0.002 to 0.005)	<0.001
	Height (cm)	0.003 (0.002 to 0.004)	<0.001
	Weight (Kg)	0.002 (0.002 to 0.003)	<0.001
**Lens thickness (mm)**	Age (years)	0.018 (0.017 to 0.019)	<0.001
	Sex (male/female)	0.026 (0.01 to 0.043)	<0.001
	Education (Each year in school)	−0.002 (−0.004 to −0.001)	0.005
	Height (cm)		NS
	Weight (Kg)	−0.001 (−0.002 to −0.001)	<0.001
**Vitreous chamber depth (mm)**	Age (years)	−0.011 (−0.015 to −0.007)	<0.001
	Sex (male/female)		NS
	Education (Each year in school)	0.009 (0.004 to −0.014)	<0.001
	Height (cm)	0.023 (0.021 to −0.026)	<0.001
	Weight (Kg)	0.004 (0.001 to −0.006)	<0.001

CI = confidence interval.

## Discussion

AL has different applications in ophthalmology, and so, we used various statistical indices such as the 95% CI, normal range, and percentiles to describe the distribution of these variables. Describing the normal range of this index can be important in the choice of formula used for intraocular lens calculation in cataract patients. The distribution of AL in the normal population in Iran has not been studied, but according to other studies (Table 6) AL varies between 22.6 mm to 24.09 mm, and the mean AL in our study falls in the midrange. The first reason for this variation could be the age range of samples, because as demonstrated here and in other studies [6,14], AL decreases with age, and in Table 6, the highest AL is seen in the 17–30 year old age group, and the lowest AL value belongs to over 70 year olds. Comparison of AL in people over 40 years in different regions shows that the index varies between 22.6 mm to 23.6 mm in this age group. On one hand, Warrier et al. [6] reported a mean AL of 22.75 mm in 60 to 69 year olds, while the 59 to 64 year old group in the study by Fotedar et al. [14] had a mean AL of 23.60 mm. Overall, much of these differences can be explained by racial and genetic differences [15]. The direct relationship between AL and height has been demonstrated in previous studies as well, [16-18] and this relationship can explain part of the differences seen in samples and populations of different heights. Environmental and life style factors, such as industrialization, which can influence the rate of near work, should be considered as well.

**Table 6:** Mean axial length, anterior chamber depth, lens thickness, and vitreous chamber depth reported in population-based studies compared to findings of the present study

**Author**	**Age (Year)**	**Place**	**AL (mm)**	**ACD (mm)**	**LT (mm)**	**VCD (mm)**
**Wong**[7]	40 to 81	Singapore	23.23	2.90	4.75	15.58
**Wickremasinghe**[4]	40-49	Mongolia	23.2	3.0	4.2	16.0
**Wickremasinghe**[4]	50-59	Mongolia	23.2	2.8	4.4	16.0
**Wickremasinghe**[4]	60-69	Mongolia	23.3	2.7	4.5	16.0
**Wickremasinghe**[4]	>70	Mongolia	23.3	2.6	4.6	16.0
**Warrier**[6]	40-49	Myanmar	22.75	3.03	4.31	15.41
**Warrier**[6]	50-59	Myanmar	22.74	2.84	4.51	15.39
**Warrier**[6]	60-69	Myanmar	22.75	2.76	4.59	15.40
**Warrier**[6]	>70	Myanmar	22.73	2.69	4.63	15.41
**Warrier**[6]	40+	Myanmar	22.76	2.82	4.51	15.43
**Shufelt**[19]	40+	United State	23.38	3.41	4.38	15.04
**Fotedar**[14]	59-64	Australia	23.60	3.20	-	-
**Fotedar**[14]	65-74	Australia	23.44	3.13	-	-
**Fotedar**[14]	75-84	Australia	23.39	3.05	-	-
**Fotedar**[14]	85+	Australia	23.23	2.89	-	-
**Jivrajka**[20]	29-95	United State	23.46	2.96	4.93	-
**He**[8]	50–59	China	23.08	2.79	4.26	-
**He**[8]	60–69	China	23.14	2.65	4.47	-
**He**[8]	70–79	China	23.08	2.60	4.64	-
**He**[8]	80–93	China	23.11	2.57	4.65	-
**He**[8]	All age	China	23.11	2.67	4.44	-
**Velez-Montoya**[21]	54.71 ± 22.33	Mexico	23.33	3.25	4.52	-
**This study**	**40-64**	Iran	**23.14**	**2.62**	**4.28**	**15.72**

AL = Axial length.

ACD = Anterior chamber depth.

LT = Lens thickness.

VCD = Vitreous chamber depth.

Mean ACD in this study was 2.62 mm. As demonstrated in Table 6, the value ranges between 2.57 and 3.41 mm. XU et al. [22] showed this value to be 2.42 mm in over 45 year old Chinese, and overall, the over 40 year olds in different studies have about 1 mm difference in ACD; lower in Asians, especially in Eastern Asians, and the highest in Los Angeles Latinos. Mean ACD in this study was relatively low. A similar observation was made in the Tehran Eye Study [23] where the mean ACD in the over 40 year old age group was between 2.50 to 2.69 mm, thus, we can hypothesize that the people of Iran have short anterior chambers. Also, considering the observations in the Chinese and Mongolians compared to Americans, the ACD can be assumed smaller in Asians.

The LT in the studied population was lower compared to available studies. (Table 6) In contrast to ACD, our findings on LT were very similar to that in Americans, and considerably lower than that in Eastern Asians, especially the Chinese. However, the variation in LT in different studies is overall less than that with ACD.

In this study, AL, ACD, and VCD shortened with age, and lens thickness increased. A similar association has been reported by He et al. [24], Foster et al. [25], Fotedar et al. [14] Jivrajka et al. [20], and Warrier et al. [6]. Although an increase in AL is expectable from infancy until adolescence, [26] it is difficult to explain the shortening of the eye after middle age years. Our first impression was that we could bring up the age cohort effect, but Gudmundsdottir et al. [27] demonstrated AL shortening in a 5 year cohort study; this finding weakens the age cohort effect hypothesis, and it seems that the AL may decrease with age due to some unknown changes, especially ocular atrophy. It must also be noted that our data come from a cross-sectional study, therefore no judgment can be made about the trend of AL changes with age and longitudinal studies are needed for a definite answer.

A decrease in ACD and increase in LT with age is another finding of this study as well as other studies [24,25].An increase in LT has been observed by Mallen et al. [9] between the ages of 17 and 40 years, as well. It has also been observed by Koretz et al. [28] in monkeys. The increase in LT with age can be attributed to the increase in protein fiber layers forming under the capsule. As mentioned, the ACD decreases with age, and in this regard, the increase in LT can be the main cause for the decrease in ACD, and this has been stated in the report by Praveen et al. [29].

All of the assessed biometrics in this study were greater in men. Results of some studies concerning these findings are shown in Table 7; all studies demonstrated higher AL and ACD values in men, while the association between LT and gender has contradicting results, and even in the study by He et al. [8], this value is reported greater in women. In studies where the VCD is assessed, this index is reported higher in men. Based on these findings, inter-gender differences in refractive errors are expected. However, most studies have demonstrated a flatter corneal curvature in men [6,8,19] and although a flatter cornea can decrease part of the myopic shift of the refraction in men, most studies have shown more myopia in men [30-32] and more hyperopia in women [33]. There seems to be a more prominent role for AL in the inter-gender difference in refractive errors compared to other ocular biometrics.

**Table 7:** Summary of some other studies concerning the association of studied parameters with gender

**Place**	**Age**	**AL (mm)**	**ACD (mm)**	**LT (mm)**	**VCD (mm)**
**Myanmar**[6]	40+	M:23.12	M: 2.86	M:4.52	M: 15.74
		F:22.54	F:2.79	F:4.5	F:15.24
**Jordan**[9]	17-40	M:23.33	M: 3.17	M:3.89	M: 16.2
		F: 22.29	F:3.21	F:3.83	F:15.93
**United State**[19]	40+	M:23.65	M: 3.48	M:4.40	M: 15.22
		F: 23.18	F:3.36	F:4.3	F:14.91
**Reykjavik**[36]	55+	M:23.74	M: 3.20	M:4.68	
		F: 23.20	F:3.08	F:4.65	
**Australia**[14]	59+	M:23.75	M: 3.16		
		F: 23.20	F:3.06		
**Norfolk, UK**[18]	48-88	M:23.80	M: 3.15		
		F: 23.29	F:3.08		
**China**[8]	50+	M:23.38	M: 2.75	M:4.32	
		F: 22.83	F:2.61	F:4.50	
**Mongolia**[4]	40+	M:23.43	M: 2.87		
		F: 23.08	F:2.77		
**This study**	**40-64**	**M:23.41**	**M:2.66**	**M:4.30**	**M:15.93**
		**F:22.95**	**F:2.58**	**F:4.26**	**F:15.58**

AL = Axial length.

ACD = Anterior chamber depth.

LT = Lens thickness.

VCD = Vitreous chamber depth.

M = male.

F = female.

This study has limitations and strengths. Since detailed results with refractive errors have already been published, [34] they are not presented here. Unlike previous studies in Iran, [32,35] myopia (with the prevalence of 38.3%) was more prevalent than hyperopia (with the prevalence of 22.1%) in this population.

Although mean AL in this sample was in the mid-range compared to other studies, but as demonstrated, this index was slightly skewed to right and had a leptokurtic distribution, and thus, part of the high prevalence of myopia in the 40–64 year old population of Shahroud could be attributed to the non-normal distribution of AL in this population.

The most important strong point is that AL and its components are studied in a large sample size of 40 to 64 year old adults, which is unprecedented. It provides valuable information from a normal Iranian sample which can add to our knowledge of the Middle Eastern population. The data can serve as a helpful guideline for diagnostic and clinical purposes. Another strong point is its being a cohort and age-related changes can be studies with more validity in the next phases. Nonetheless, longitudinal studies are suggested in different populations, especially on younger samples while the eye undergoes changes.

## Conclusions

In this report, the distribution of AL and its components are described in a general 40–64 years old Iranian population for the first time. Mean AL in this study, compared to studies conducted [4-12,14-18,20-26,36] in Asia and America, was in the midrange; however, examining its components showed a lower mean ACD and LT. Except for LT which increased with age, all other parameters decrease with age. Also, the studied biometrics were all higher in men compared to women.

## Competing interests

The author declares that they have no competing interests.

## Authors’ contributions

HH, MHE, MS and AF conceived and designed the study and contributed in preparation of the study protocol and were involved in data collection supervision. HH, MK, and AF participated in study design, performed the statistical analyses and drafted the manuscript. MM, MS and TA contributed in the conceptualization of the paper and the statistical analyses and critically revised the manuscript. All authors read and critically revised the manuscript and approved the final draft.

## Pre-publication history

The pre-publication history for this paper can be accessed here:

http://www.biomedcentral.com/1471-2415/12/50/prepub
